# Sialoglycan recognition is a common connection linking acidosis, zinc, and HMGB1 in sepsis

**DOI:** 10.1073/pnas.2018090118

**Published:** 2021-03-03

**Authors:** Shoib S. Siddiqui, Chirag Dhar, Venkatasubramaniam Sundaramurthy, Aniruddha Sasmal, Hai Yu, Esther Bandala-Sanchez, Miaomiao Li, Xiaoxiao Zhang, Xi Chen, Leonard C. Harrison, Ding Xu, Ajit Varki

**Affiliations:** ^a^Department of Medicine, University of California San Diego, La Jolla, CA 92093;; ^b^Department of Cellular and Molecular Medicine, University of California San Diego, La Jolla, CA 92093;; ^c^Glycobiology Research and Training Center, University of California San Diego, La Jolla, CA 92093;; ^d^Department of Chemistry, University of California, Davis, CA 95616;; ^e^The Walter and Eliza Hall Institute of Medical Research, Parkville, VIC 3052, Australia;; ^f^Department of Medical Biology, University of Melbourne, Parkville, VIC 3010, Australia;; ^g^Department of Oral Biology, School of Dental Medicine, University at Buffalo, The State University of New York, Buffalo, NY 14214

**Keywords:** sialic acid, Neu5Ac, COVID-19, cytokine storm, HMGB1

## Abstract

Blood pH is tightly maintained between 7.35 and 7.45, and acidosis (pH <7.3) indicates poor prognosis in sepsis, wherein lactic acid from anoxic tissues overwhelms the buffering capacity of blood. Poor sepsis prognosis is also associated with low zinc levels and the release of High mobility group box 1 (HMGB1) from activated and/or necrotic cells. HMGB1 added to whole blood at physiological pH did not bind leukocyte receptors, but lowering pH with lactic acid to mimic sepsis conditions allowed binding, implying the presence of natural inhibitor(s) preventing binding at normal pH. Testing micromolar concentrations of divalent cations showed that zinc supported the robust binding of sialylated glycoproteins with HMGB1. Further characterizing HMGB1 as a sialic acid-binding lectin, we found that optimal binding takes place at normal blood pH and is markedly reduced when pH is adjusted with lactic acid to levels found in sepsis. Glycan array studies confirmed the binding of HMGB1 to sialylated glycan sequences typically found on plasma glycoproteins, with binding again being dependent on zinc and normal blood pH. Thus, HMGB1-mediated hyperactivation of innate immunity in sepsis requires acidosis, and micromolar zinc concentrations are protective. We suggest that the potent inflammatory effects of HMGB1 are kept in check via sequestration by plasma sialoglycoproteins at physiological pH and triggered when pH and zinc levels fall in late stages of sepsis. Current clinical trials independently studying zinc supplementation, HMGB1 inhibition, or pH normalization may be more successful if these approaches are combined and perhaps supplemented by infusions of heavily sialylated molecules.

## Significance

Sepsis is a condition wherein a microbial infection leads to life-threatening systemic hyperactivation of innate immunity. Blood pH is normally maintained tightly between 7.35 and 7.45, and lactic acidosis with a pH <7.3 indicates a poor prognosis in sepsis, also associated with low zinc levels. Release of HMGB1 from activated and/or necrotic tissues plays a pivotal role in triggering the proinflammatory cascade of late sepsis. Using an in vitro whole-blood assay, we observed that HMGB1 cannot mediate proinflammatory activity at physiological pH and zinc concentrations. This is due to zinc-dependent association of HMGB1 with sialoglycoproteins, thereby preventing its binding with proinflammatory receptors. Thus, a drop in pH and zinc concentration in sepsis can release sequestered HMGB1 and trigger the inflammatory cascade.

The pH of body fluids in healthy individuals spans a very broad range in different tissue types and organs, ranging from pH 1.5 (stomach contents) to 8.0 (urine). Human cells in tissue culture can also tolerate a wide range of pH values. In contrast, blood pH is tightly regulated between 7.35 and 7.45 (1), and departure out of this range (acidosis or alkalosis) can be very detrimental. For example, in the recent COVID-19 pandemic, 30% of nonsurvivors had acidosis, compared to 1% among survivors (2). Acidosis in sepsis is partly due to lactic acid release from anoxic tissues, which overwhelms the buffering capacity of circulating blood (3). A “cytokine storm” of proinflammatory mediators in sepsis triggers a cascade of destructive outcomes such as multiple organ failure (4–8) as currently seen in severe cases of COVID-19 infection (9). The mechanisms underlying lethality associated with low blood pH are not clear but include low zinc levels and release from apoptotic or necrotic cells of High mobility group box 1 (HMGB1), a damage-associated molecular pattern (DAMP) defined as one of the late mediators of sepsis, further up-regulating many other proinflammatory cytokines (10–12). Importantly, a recent study indicates that HMGB1 levels are strongly associated with mortality in patients infected with severe acute respiratory syndrome coronavirus 2 (SARS-CoV-2) (13). Here we show that sialylated plasma glycoproteins bind HMGB1 to suppress its ability to promote inflammatory responses in a zinc- and pH-dependent manner. Besides providing an explanation for the very tight regulation of blood pH, these findings provide an avenue for developing a new therapeutic strategy for treating sepsis.

## Results

### Mimicking Lactic Acidosis Ex Vivo in Hirudin-Anticoagulated Whole Blood.

In vivo studies of acidosis and sepsis involve many complex factors and interactions. On the other hand, ex vivo reconstitution of purified blood components can result in artifacts; for example, neutrophils get activated when separated away from erythrocytes and plasma (14). To study the significance of tightly regulated blood pH ex vivo, we sought to create a whole-blood system mimicking lactic acidosis. Conventional anticoagulation with ethylenediaminetetraacetic acid (EDTA) or citrate abrogates divalent cation functions, and heparin has many biological effects independent of anticoagulation. We have previously shown that the leech protein hirudin can be used for whole-blood anticoagulation in vitro (15). When lactic acid was added to freshly collected hirudin-anticoagulated whole blood, the pH first rose until a concentration of about 1 mM lactic acid was reached. Further addition then caused a sharp drop in blood pH (*SI Appendix*, Fig. S1). Such an initial rise in blood pH followed by a subsequent drop is seen in patients with sepsis (16). To further develop this model, we introduced HMGB1, a DAMP (17–19) associated with poor prognosis in late sepsis (20, 21).

### Neutrophils in Whole Blood Are Activated by HMGB1 at Low pH due to Better Binding, and Activation Is Attenuated with an HMGB1-Blocking Antibody.

**Fig. 1.**
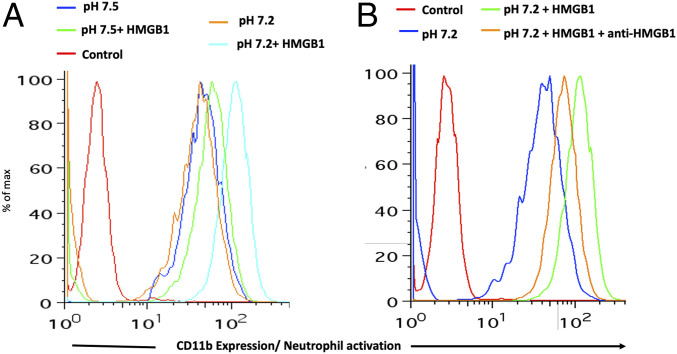
Mimicking sepsis by adding lactic acid to whole blood triggers activation of neutrophils by HMGB1, which is partially attenuated by an HMGB1-blocking antibody. CD11b expression was determined by flow cytometry after incubating whole blood with/without HMGB1 (1 µg/mL). (*A*) Neutrophils are activated when incubated with HMGB1 in whole blood at pH 7.2 (chromatograms: red, isotype control; blue, whole blood at pH 7.5; orange, whole blood at pH 7.2; green, whole blood at pH 7.5 with HMGB1; cyan, whole blood at pH 7.2 with HMGB1). (*B*) Activation is partially attenuated with an HMGB1-blocking antibody (50 µg/mL) (chromatograms: red, isotype control; blue, whole blood at pH 7.2; green, whole blood at pH 7.2 with HMGB1; orange, whole blood at pH 7.2 with HMGB1 and an HMGB1-blocking antibody). The result is representative of three independent experiments on the blood of a healthy individual.

**Fig. 2.**
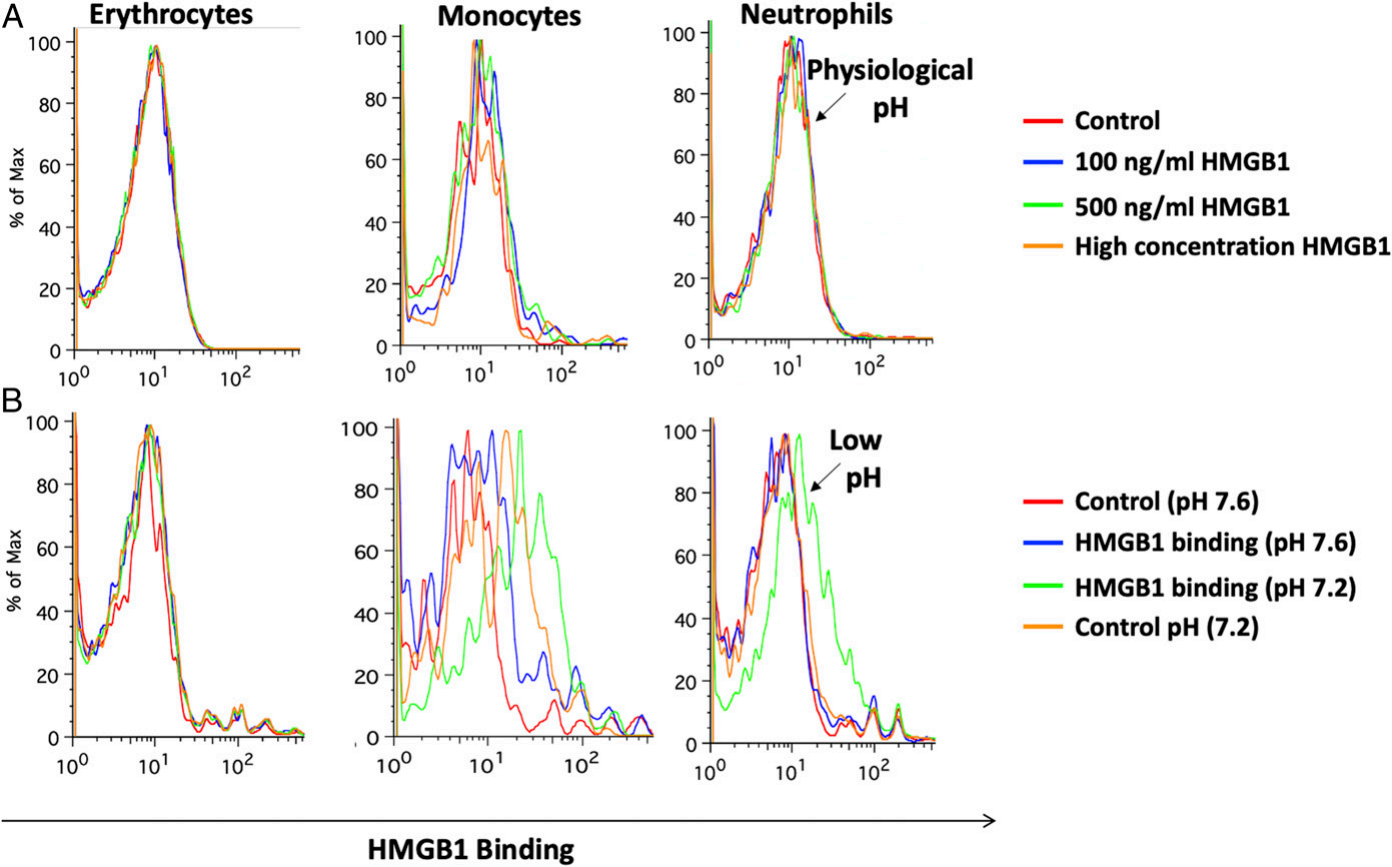
Mimicking sepsis by adding lactic acid to whole blood triggers binding of HMGB1 to leukocytes. (*A*) Ability of HMGB1 to bind to different cell types of the blood (erythrocytes, monocytes, and neutrophils) was determined by using different concentrations (100 ng/mL, 500 ng/mL, and 5 µg/mL) of HMGB1 at physiological conditions. (*B*) Different cell types of blood were used for binding with HMGB1 (100 ng/mL) at physiological and lower pH (pH 7.2, adjusted with lactic acid). The data show one representative flow cytometry histogram result of two separate blood samples assayed.

Interaction of HMGB1 with Toll-like receptors (TLRs) during sepsis is well-documented (22). The proinflammatory activity of HMGB1 is due to binding to targets such as TLR-2, TLR-4, TLR-9, and RAGE that are expressed on leukocytes and endothelial cells (23, 24). We, therefore, introduced exogenous HMGB1 into our whole-blood acidosis model and tracked CD11b expression on neutrophils, as a sensitive marker of activation triggered by HMGB1. Increased neutrophil activation was noted when HMGB1 was incubated with whole blood at low pH as compared to physiological pH (Fig. 1*A*). This effect was partially attenuated by adding HMGB1 blocking antibody (Fig. 1*B*). It is noteworthy that the pH itself does not have an impact on the activation status of the neutrophils, as there was no difference in the expression of CD11b at pH 7.2 and pH 7.5 (Fig. 1*A*). Enhanced activation at low pH coincides with increased HMGB1 binding to neutrophils and monocytes (compare Fig. 2 *A* and *B*). Thus, physiological blood pH limits interaction of HMGB1 with leukocyte receptors, suggesting natural inhibitor(s) of HMGB1 interaction in blood. Looking for candidate inhibitors, we noted earlier evidence that HMGB1 can interact with CD24 and CD52, two heavily sialylated proteins (25, 26) in a trimolecular complex with Siglec-10, a known sialic acid-binding protein. CD52-Fc bound specifically to the proinflammatory Box B domain of HMGB1, and this, in turn, promoted binding of the CD52 N-linked glycan sialic acid with Siglec-10 (26). Furthermore, sialidase treatment abolished CD52 binding to HMGB1, indicating that HMGB1 might be a sialic acid-binding lectin. Since normal blood plasma contains ∼2 mM sialic acid attached to glycans on plasma proteins (27), we hypothesized that the unknown natural inhibitor might be the sialome (the total sum of all sialic acids presented on plasma glycoproteins).

### Among Divalent Cations, Only Zinc Supported the Robust Binding of HMGB1 with Sialylated Glycoproteins at Physiological pH.

**Fig. 3.**
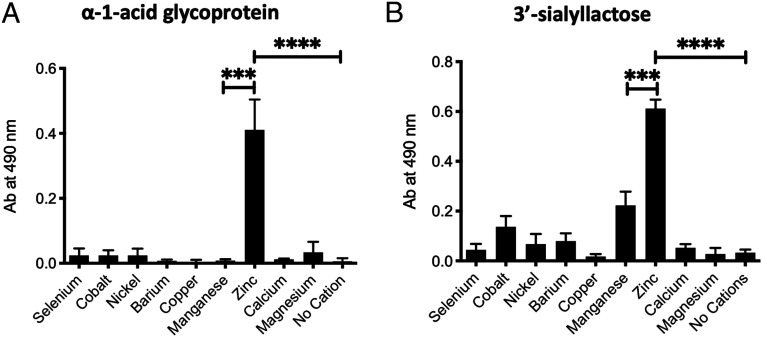
Among divalent cations, only zinc supported robust binding of HMGB1 with sialylated glycoproteins.: (*A* and *B*) Multiple divalent cations (labeled in the figure) were used individually in the binding buffer, each at a concentration of 500 µM and binding with human α1-acid glycoprotein and 3′-sialyllactose was determined at pH 7.5 using ELISA. The experiments were performed in triplicate where data show mean ± SD. The replicates mentioned were technical replicates and a *t* test was performed to find the statistical significance. The following *P* values were observed: ***P < 0.001 and ****P < 0.0001.

The binding buffer used in prior HMGB1 studies included millimolar concentrations of manganese cation (Mn^2+^), a feature likely carried over from the unrelated function of nuclear HMGB1 binding to DNA. Looking at earlier studies of the interaction of HMGB1 with CD24 and CD52, we noticed that all those experiments were performed in a buffer containing millimolar Mn^2+^ concentrations (25, 28–30). These concentrations were very high in comparison with the physiological levels of Mn^2+^ in the blood (4 to 15 µg/L). We predicted that there might be other divalent cation(s) that are better cofactor(s) for HMGB1 and facilitate its binding with sialic acids. Indeed, upon testing micromolar concentrations of many divalent cations, we found that only zinc cation (Zn^2+^) supported robust binding with sialylated glycoproteins (Fig. 3*A*). We tested α_1_-acid glycoprotein and 3′-sialyllactose as binding partners for HMGB1 in the presence of different cations and again found that only Zn^2+^ facilitated binding. There was a modest binding of 3′-sialyllactose with HMGB1 in the presence of Mn^2+^, but the robust binding was only seen with Zn^2+^-containing buffer (Fig. 3*B*).

### Replacing Plasma with Buffer at Physiological pH Allows HMGB1 to Activate Neutrophils, Suggesting Sequestration by Plasma Sialoglycoproteins.

**Fig. 4.**
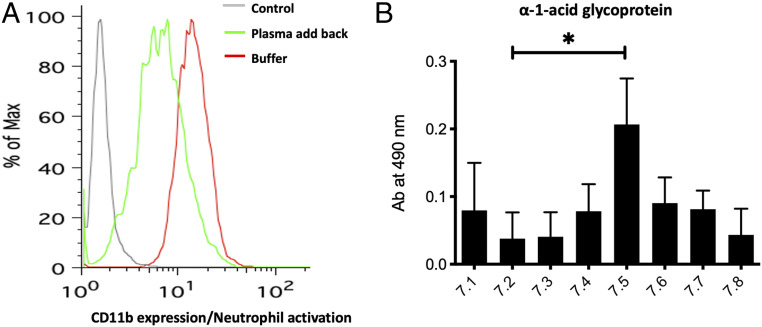
Replacing plasma with a buffer at physiological pH allows HMGB1 to activate neutrophils. (*A*) One milliliter of blood was drawn from a healthy individual and spun down. The plasma was replaced either with Hepes buffer containing zinc (500 µM of Zn^2+^) or with the plasma that had been removed. Under both conditions HMGB1 was added and incubated and CD11b expression as a marker of neutrophil activation was measured (representative image of assays on two different blood samples). (*B*) The binding of HMGB1 to α1-acid glycoprotein was checked with a binding buffer using different pH ranging from 7.1 to 7.8. The following *P* value was observed: *P < 0.05.

We next asked which whole-blood components were preventing neutrophil activation under physiological conditions. Hirudin-anticoagulated whole blood at physiological pH was spun down and plasma either replaced with Hepes buffer (pH 7.5) supplemented with Zn^2+^ or with the same plasma that had been removed. After incubating with HMGB1, neutrophils were in a more activated state when incubated in the buffer as compared to when plasma was added back (Fig. 4*A*). Part of this effect was mediated by the exogenously added HMGB1 (*SI Appendix*, Fig. S2). Independent studies have shown that HMGB1 binds to sialic acid on glycoproteins (26, 31) and we posited that the ∼2 mM bound sialic acid present on plasma glycoproteins might lead to sequestration of HMGB1 under physiological condition. We also tested the effect of pH on the binding of HMGB1 to α_1_-acid glycoprotein and found that optimal binding was at physiological pH, with less binding at pH 7.2 with buffer containing Zn^2+^ (Fig. 4*B*).

### Sialoglycan Array Studies of HMGB1 Confirm That It Is a Sialic Acid-Binding Lectin with Optimal Binding at Physiological Blood pH in the Presence of Zinc Cations.

**Fig. 5.**
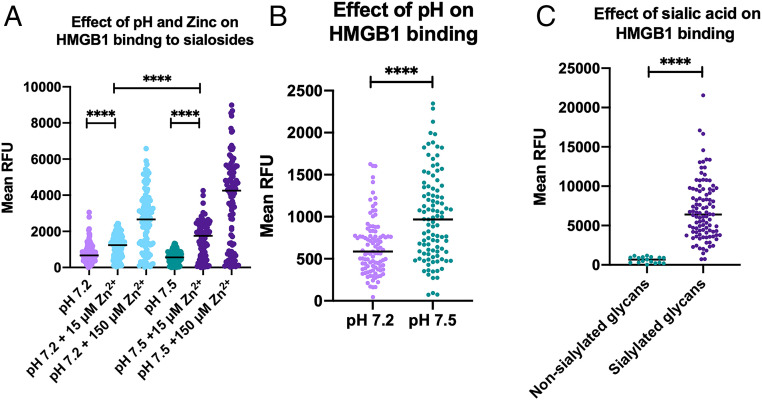
Sialoglycan array studies of HMGB1 confirm that it is a sialic acid-binding lectin with optimal binding at physiological blood pH in the presence of zinc. (*A*) The sialoglycan array was performed to test the binding of HMGB1 with multiple sialylated probes. The binding buffer used for the assay either contained zinc and was pH 7.5; no zinc, pH 7.5; with zinc, pH 7.2; and no zinc at pH 7.2. The concentration of zinc used was 15 µM and 150 µM (representative image of a single experiment. Wilcoxon matched pairs signed rank test used. ****P < 0.0001). (*B*) Additional microarray experiments with 500 µM zinc further resolve the pH-dependent binding difference (representative image of the mean of two experiments. Unpaired *t* test with Welch’s correction used to compare the two groups. ****P < 0.0001). (*C*) The difference in the level of HMGB1 binding to sialosides and nonsialosides at physiological pH in the presence of 500 µM zinc (representative image of a single experiment where zinc was used at a concentration of 500 µM. Kolmogorov–Smirnov test used. ****P < 0.0001).

We previously reported a sialoglycan microarray platform used to identify, characterize, and validate the Sia (sialic acid)-binding properties of proteins, lectins, and antibodies (32–34). After identifying Zn^2+^-dependent HMGB1 binding to sialoglycoproteins, we next investigated the ability of HMGB1 to bind with multiple sialoglycans abundantly found in plasma proteins. We performed sialoglycan array studies of HMGB1 under four different conditions: 1) at physiological pH with Zn^2+^, 2) at physiological pH without Zn^2+^, 3) at pH 7.2 with Zn^2+^, and 4) at pH 7.2 without Zn^2+^. These array studies further confirmed the binding of HMGB1 with multiple sialylated glycan sequences that are typically found on plasma glycoproteins, in pH- and Zn^2+^-dependent fashion (Fig. 5 *A* and *B*, respectively). Additionally, we checked the binding of HMGB1 to sialic acids in sialoglycan microarray using 0, 15, and 150 µM concentrations of Zn^2+^ and observed a dose-dependent effect (Fig. 5*A*). This assay showed the relevance of Zn^2+^ in this binding phenomenon at a physiological concentration (∼100 µM). There was an abundant binding of HMGB1 to sialylated probes in glycan array at physiological pH compared to lower pH (Fig. 5*B*). On resolving the binding of HMGB1 at physiological pH and in the presence of zinc, the binding on the microarray was exclusively to sialylated glycans confirming our findings (Fig. 5*C*). A heat-map representation of all these findings and HMGB1 binding to individual glycosides is provided in *SI Appendix*, Figs. S5 and S6, respectively.

### Heparin, a Previously Known Anionic Glycan Binding Partner of HMGB1, Does Not Exhibit pH Sensitivity, and Zn^2+^ Only Partially Facilitates Binding.

HMGB1 is known to bind heparin, a heavily sulfated glycan carrying many negatively charged groups (35, 36). We checked the binding of HMGB1 with heparin at different pH values and found that unlike binding with Sia it was not pH-sensitive (*SI Appendix*, Fig. S3*A*). Moreover, there was appreciable baseline binding of HMGB1 with heparin that only increased partially with Zn^2+^ supplementation (*SI Appendix*, Fig. S3*B*). These data indicate that the binding of heparin and sialic acid are very different. The B Box of HMGB1 that mediates sialic acid binding (26) has three arginine residues (26) that might be involved in sialic acid recognition. We made single mutants of arginine residues at positions 97, 110, and 163. When we checked the sialic acid binding, we could not find any difference between either of the mutants and wild-type (WT) HMGB1 (*SI Appendix*, Fig. S4). We suspect other positively charged residues and/or multiple arginines mediate sialic acid binding.

Based on all the evidence we have gathered, we believe the chances are that heparan sulfate (HS) will not affect how HMGB1 interacts with sialylated plasma proteins. We have actually tested the binding of an HS-binding-deficient quintuple mutant of HMGB1 (35) to sialyated plasma protein and found that the mutant showed binding comparable to WT HMGB1 (*SI Appendix*, Fig. S8). This result suggests that the HS-binding site and sialic acid (SA)-binding site are not overlapping, and that HMGB1 could simultaneously bind both glycans. However, under pathological conditions the soluble HS content in plasma could rise. The soluble HS might have an impact on how HMGB1 binds to myeloid cell surfaces because it could compete with heparan sulfate proteoglycans (HSPG) expressed by myeloid cells for HMGB1 binding.

## Discussion

Here we report one plausible explanation for the tight regulation of blood pH between 7.35 and 7.45, showing that even a slight reduction to pH 7.2 abolishes the zinc-dependent sequestration of HMGB1 potentially by plasma sialoglycoproteins, releasing it to bind to activating receptors on neutrophils. HMGB1 was originally discovered in the cell nucleus (37–40), playing a role in DNA bending, replication, and transcription (41, 42). Much later, HMGB1 was found to be passively or actively released in conditions like sepsis, leading to inflammation (21, 41, 43), that is, it is a DAMP (44). HMGB1 retention inside the nucleus is dictated by conserved lysine residues (45). Inflammatory stimuli trigger acetylation of these lysine residues and trafficking of HMGB1 to the cytosol, and eventually to the extracellular space. The different domains of HMGB1 are Box A, Box B, and an acidic tail. While Box A and Box B possess many arginine and lysine residues, the acidic tail is enriched with glutamic and aspartic acid residues. Box B is proinflammatory, whereas Box A behaves like an antagonist and mimics an anti-HMGB1 antibody (26, 46).

While tumor necrosis factor α and interleukin 1β are released early during sepsis, HMGB1 is a late mediator expressed only after about 24 h and remains at elevated levels before death occurs (47). Many preclinical studies show protection against sepsis upon injection of blocking antibodies of HMGB1 or injection of Box A protein (48). The proinflammatory activity of HMGB1 is well studied. However, the antiinflammatory activity of HMGB1 also has been documented in multiple studies (49–51). Recently, it was shown that HMGB1 binds soluble CD52 and this complex binds with Siglec-10 on T cells, leading to SHP-1 (phosphatase) recruitment that dephosphorylates LCK and Zap70, thus activating an antiinflammatory cascade (26, 52). In addition, haptoglobin (49), C1q, and TIM3 also show antiinflammatory activity of HMGB1 (50, 51).

In this study, we found that in whole blood at physiological pH there is no interaction of HMGB1 with its receptors on leukocytes. Surprisingly, when we lowered the pH using lactic acid (to mimic lactic acidosis, a characteristic feature of sepsis) the interaction was restored. Furthermore, the high concentration of sialic acids in plasma glycoproteins was found to be the likely inhibitor of interactions between HMGB1 and TLRs. We further characterized the role of HMGB1 as a sialic acid-binding lectin and found that zinc is a required cofactor. Moreover, we confirmed all our findings with lipopolysaccharide-free HMGB1 and used a glycan array that detected the binding of HMGB1 with several sialic acid probes (*SI Appendix*, Table S1) in a pH- and zinc-dependent manner.

**Fig. 6.**
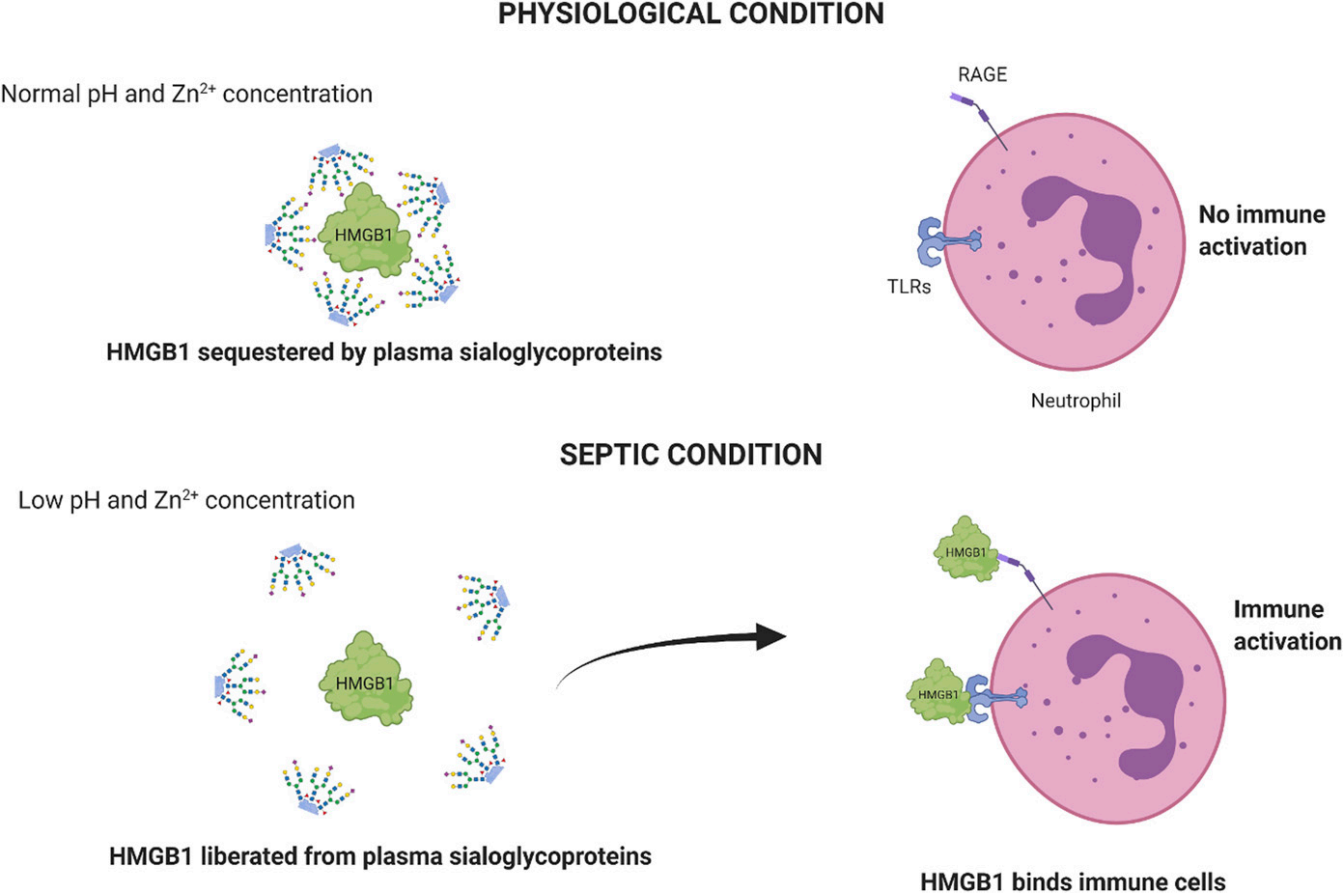
Proposed model of sequestration of HMGB1 by sialoglycoproteins to prevent HMGB1 binding to receptors on leukocytes. A schematic showing the binding of HMGB1 to sialic acid under physiological pH (*Upper*) and binding to leukocyte receptors at low pH (*Lower*).

In this study, we used CD11b as an activation marker of neutrophils. This is a well-established method to study the activation/inactivation of neutrophils with the treatment (53–55). There is an increase in CD11b levels upon activation of neutrophils. We observed an increase in the CD11b upon reduction in pH accompanied by the addition of HMGB1. Taken together, our findings lead us to propose that under physiological conditions (pH 7.35 to 7.45) and normal zinc concentrations there is a potent binding of HMGB1 with plasma sialoglycoproteins (Fig. 6, *Upper*). Under septic conditions, drops in pH and zinc concentration decrease interactions between HMGB1 and plasma sialoglycoproteins, leading to the liberation of HMGB1 to bind with TLRs, to enhance inflammation (Fig. 6, *Lower*). Therefore, proinflammatory and antiinflammatory activities of HMGB1 are the two sides of the same coin and are dependent on the different physiological conditions. While the proinflammatory role of HMGB1 is very well studied, recent studies have reported an antiinflammatory role for HMGB1 (25, 50–52). The exact mechanism that enables HMGB1 to switch from its proinflammatory to antiinflammatory role, and vice versa, is not very well described. One factor known to enable its switch from being proinflammatory to antiinflammatory is its oxidative state. The disulfide form of HMGB1 is proinflammatory, and the sulfonate form is involved in the resolution of inflammation (56–58). In the current study, we have identified another mechanism by which HMGB1 switches from its proinflammatory to antiinflammatory role in a pH- and zinc-dependent manner. Sepsis is characterized by a decrease in pH and zinc concentration of the blood. We hypothesize that under physiological conditions HMGB1 binds with sialoglycoproteins of blood, keeping it in a quiescent state. During sepsis, the drop in pH and zinc concentration of the blood leads to disruption of HMGB1’s binding with sialic acid, enabling the free HMGB1 to bind with TLRs and RAGE present on immune cells and the endothelium. This activates a cascade of the inflammatory response, which if untreated might lead to multiple organ failure or even death.

The physiological concentration of zinc in the blood is reported to near 100 µM (59, 60). Levels of zinc in plasma and serum are the most commonly used parameters to determine the zinc concentration in the blood. The differences in values of zinc concentration between serum and plasma in the same individual have been attributed to 1) different procedures for collecting and separating serum and plasma and 2) the delay in processing times for serum and plasma. When both these variables were controlled for, no differences were observed in plasma and serum zinc concentration (60). There appears to be little variation in zinc concentration in the blood between different individuals. The values of zinc concentration in the blood from different studies are comparable (60). This value is of the same order of magnitude as the zinc concentration that was used in our study.

Also consistent with our hypothesis are the findings that survival in mouse models of sepsis can be improved by infusion of soluble CD52 (61), and that the sialic acid-binding feature of HMGB1 is restricted to the disulfide form of HMGB1 (26), which is expected to be formed when the cytosolic reduced form is released into the oxidizing environment of the bloodstream. We suggest that the potent proinflammatory effects of HMGB1 are normally kept in check via sequestration by plasma sialoglycoproteins at physiological pH and zinc levels and are triggered when pH and zinc levels fall in the late stages of sepsis. In this regard, it is notable that the acute phase response to inflammation results in high production of hypersialylated molecules such as α1-acid glycoprotein from the liver and endothelium, which may then act as a negative feedback loop (62–65). Current clinical trials that are independently studying zinc supplementation (ClinicalTrials.gov Identifier: NCT01328509 NCT02130388) or pH normalization (NCT03530046) may be more successful if these approaches are combined, and perhaps supplemented by infusions of heavily sialylated molecules like CD52. Additionally, studies evaluating plasma exchange in subjects with septic shock (example NCT03366220) may show superior efficacy if supplemented with zinc infusions and pH correction. Preclinical studies are presently evaluating the function of a blocking anti-HMGB1 antibody (66). We performed our assays with HMGB1 purchased from HMG Biotech, also produced it in *Escherichia coli* and finally confirmed findings using HMGB1 expressed in 293 FreeStyle cells. In order to recapitulate the characteristics of HMGB1 in septic conditions, we used the disulfide-linked form in all our assays. Future studies should address whether other posttranslational modifications such as acetylation, methylation, phosphorylation, or oxidation have any further effect on HMGB1’s propensity to bind sialic acids.

Many studies have shown that zinc is protective against sepsis (67–69). One of these studies reports serum zinc concentration in sepsis patients of around 4 µM, compared to ∼11 µM in healthy individuals. Additionally, blood zinc levels usually decrease during inflammation because it is sequestered to the nucleus where it is required as a cofactor for expression of proinflammatory genes and proteins (67, 70, 71). Thus, lowering of zinc level in the blood is detrimental. The mechanism of action for the antiinflammatory effect of zinc is also extensively studied. These include impact on the microbiome, lowering of nuclear factor κB levels, chemotaxis and phagocytosis by immune cells, antioxidative stress, and adaptive immune response (67).

In this regard, it is notable that a recent study also shows the role of zinc, pH, and ionic strength on the oligomerization of HMGB1 (72). We did not investigate any role of zinc or pH on the structural changes or oligomerization of HMGB1. It seems that at particular pH and zinc concentration a positively charged residue of HMGB1 is exposed for binding with sialic acid. This residue may not be surface-available at lower pH and low zinc concentration. In this study, we could not pinpoint the critical residue that is important for sialic acid binding.

HMGB1 has been reported to bind many ligands, some of which are highly negatively charged molecules such as heparin/heparan sulfate (35). We wanted to determine if the interaction of HMGB1 with sialic acid, which is also negatively charged, is a generic electrostatic charge-based interaction. Upon testing with heparin, we found that while HMGB1 did bind with heparin it did not show any pH dependency. Moreover, binding was only partially enhanced in the presence of zinc. This shows that a different set of amino acid(s) might be required for binding to heparin and sialic acid. Notably, under physiological conditions sialic acid is present in the blood, but the concentrations of other anionic glycans (heparan sulfate, hyaluronic acid, etc.) are low.

Our findings, if confirmed in randomized clinical trials, have broad implications in the management of sepsis and possibly other types of acidosis. Sepsis is a significant cause of mortality, with a recent study implicating it as the cause of twice as many deaths as earlier estimated (73). These findings are of particular importance in light of the present COVID-19 pandemic/survivorship in these patients. Acute respiratory distress syndrome, a deadly complication of the SARS-CoV-2 and SARS-CoV-1, has been linked with HMGB1 production (74–76). Recent articles suggest a potential link between HMGB1 and the pathogenesis of COVID-19 (77, 78). A recent study showed that HMGB1 strongly correlates with mortality in COVID-19 patients (13). Additionally, another recent study showed 100% of COVID-19 nonsurvivors had sepsis and 30% of these had acidosis (2). While the Surviving Sepsis Campaign does not suggest the use of convalescent plasma in critically ill patients (79), the Food and Drug Administration has approved its use as an investigational new drug. A small study of five critically ill COVID-19 patients treated with convalescent plasma showed improvements in sepsis-related Sequential Organ Failure Assessment scores (80). A ClinicalTrials.gov search for “COVID” and “convalescent plasma” on 6 April 2020 yielded nine results of trials ranging from phase 1 to phase 3. While the circulating antibodies are likely to be beneficial on their own, the HMGB1-sequestering properties of plasma sialoglycoproteins may also contribute to suppressing the “cytokine storm.” These effects are likely to be further enhanced if plasmapheresis is supplemented with aggressive pH correction and zinc supplementation.

To the best of our knowledge, this is one of the first studies where the whole-blood acidosis method has been used to study sepsis. We have previously shown that sialome of red blood cells (RBCs) can inhibit the activation of neutrophils partly due to interaction with Siglec-9 (14). We believe there are several other factors that can modulate the activation of neutrophils. The establishment of assays for the identification of such factors will be the focus of future studies.

## Materials and Methods

### Enzyme-Linked Immunosorbent Assay for Binding of HMGB1 with α_1_-Acid Glycoprotein or 3′-Sialyllactose.

Five hundred nanograms to 1 µg of HMGB1 recombinant protein (HMG Biotech) diluted with the binding buffer (20 mM Hepes, 150 mM NaCl, and 500 µM ZnCl_2_) was immobilized by applying on a 96-well flat-bottom plate (9018; Corning Costar) and incubating overnight at 4 °C. The wells were washed thrice with 200 µL of binding buffer per well, followed by blocking with 150 µL of 5% bovine serum albumin (BSA) (prepared in binding buffer). The plate was then incubated at room temperature (RT) for 1 h with shaking. The blocking solution was removed by flicking plate and tapping at a dry paper towel. Then, 1 µg/well of α_1_-acid glycoprotein (112150; Calbiochem-Behring) or 3′-sialyllactose-PAA-biotinylated (01-038; Glycotech), diluted in binding buffer, was applied on every well except the secondary antibody control wells which were left with only binding buffer. The plate was incubated 1 to 2 h at RT on the shaker. The solution was removed and wells were washed thrice with 200 µL binding buffer per well. The secondary antibody (Streptavidin-HRP [horseradish peroxidase], ab7403-500; Abcam) was applied at a dilution of 1:20,000 in binding buffer and the plate was incubated for 1 h at RT with shaking. Then, *O*-phenylenediamine (OPD)-based substrate solution for HRP was prepared by adding 5 mg of OPD and 25 µL of 30% H_2_O_2_ to 15 mL of citrate-PO_4−_ buffer. One hundred forty microliters of OPD substrate solution was added to each well and incubated in the dark until color development. Upon color development, the reaction was stopped using 40 µL of 2N H_2_SO_4_ and the absorbance was acquired at 490 nm with a plate reader. For the enzyme-linked immunosorbent assay (ELISA) with different divalent cations, the binding buffer was prepared using the particular cation-containing salt instead of ZnCl_2_. Each incubation and wash was performed using the respective binding buffer.

### Hirudin-Anticoagulated Whole-Blood Assays.

Informed consent was obtained from healthy individuals after a full protocol was approved by the University of California San Diego Human Research Protection Programs Institutional Review Board. Venous blood was collected in hirudin-coated tubes (NC1054637; Thermo Fisher). Hirudin was chosen as the anticoagulant as EDTA and heparin interferes with normal bioprocesses (chelation by EDTA and binding to and modulating cell-surface proteins by heparin). The pH of blood, when measured at the start of various assays, varied between 7.5 and 7.6 and is referred to as the “physiological” pH.

### Flow Cytometry Analysis for HMGB1 Activation of/Binding to Leukocytes.

To test for neutrophil activation, 100 µL of whole blood was incubated with 1 µg/mL of HMGB1 for 30 min at 37 °C. In this study, we used the side and forward scatter characteristics in flow cytometer to gate for live neutrophils (*SI Appendix*, Fig. S7). This is a routine method to identify the granulocytes, monocytes, and lymphocytes in flow cytometry. Several other published studies have utilized this method for gating neutrophils (81–83). CD11b expression was measured by flow cytometry as described earlier (14, 84). Blocking with an anti-HMGB1 antibody (Clone 3E8, 651402; BioLegend) was performed with 50 µg/mL antibody as described earlier (66). For plasma add-back studies, whole blood was spun down at 500 × *g* for 5 min and replaced with Hepes buffer supplemented with 500 µM ZnCl_2._ Binding assays were performed with 500 µL of whole blood. The required amount of HMGB1 (0, 100, 500, or 5,000 ng/mL) was added to 500 µL of blood and incubated at 37 °C for 60 min with rotation. After centrifuging at 600 × *g* for 5 min, the cells were washed with 1 mL of phosphate-buffered saline (PBS) and finally resuspended in 100 µL of FACS buffer (1% BSA in PBS with Ca^2+^/Mg^2+^) with anti-HMGB1 antibody (10 µg/mL, 651402; BioLegend). The cells were incubated at 4 °C for 30 min on ice and were washed with 1 mL PBS (containing Ca^2+^/Mg^2+^). The cells were subsequently resuspended in 100 µL of FACS buffer with a secondary anti-mouse-APC antibody (405308; BioLegend). The cells were incubated at 4 °C for 30 min on ice and washed with PBS as before. Ten microliters was taken from each sample for RBC analysis and the rest of the sample was fixed with 4% paraformaldehyde and incubated on ice for 20 min. The sample was then washed with PBS and subsequently treated with ACK lysis buffer (A10492-01; Gibco) to perform analysis of RBCs. The sample was washed and resuspended in 500 µL of FACS buffer. In the forward and side scatter profile, monocytes and neutrophils were gated for the analysis. For gating of monocytes forward and side scatter pattern was used (*SI Appendix*, Fig. S7). Histograms were created using FlowJo and visually inspected for trends in binding and activation. No statistical analysis was run.

### Glycan Array Analysis for the Binding of HMGB1 with Sialic Acids.

Chemoenzymatically synthesized sialyl glycans were quantitated utilizing DMB (1,2-diamino-4,5-methylenedioxybenzene) high-performance liquid chromatography analysis and were dissolved in 300 mM sodium phosphate buffer (pH 8.4) to a final concentration of 100 µM. ArrayIt SpotBot Extreme was used for printing the sialoglycans on NHS-functionalized glass slides (PolyAn 3D-NHS slides, PO-10400401; Automate Scientific). Purified mouse anti-HMGB1 antibody (651402, lot B219634; BioLegend) and Cy3-conjugated goat anti-mouse IgG (115-165-008; Jackson ImmunoResearch) were used. Fresh Hepes buffer (20 mM Hepes and 150 mM NaCl ± 500 µM ZnCl_2_) was prepared immediately before starting the microarray experiments.

The method described in ref. 34 was adapted to perform the microarray experiment. Each glycan was printed in quadruplet. The temperature (20 °C) and humidity (70%) inside the ArrayIt printing chamber were rigorously maintained during the printing process. The slides were left for drying for an additional 8 h. Printed glycan microarray slides were blocked with prewarmed 0.05 M ethanolamine solution (in 0.1 M Tris⋅HCl, pH 9.0), washed with warm Milli-Q water, dried, and then fitted in a multiwell microarray hybridization cassette (ArrayIt) to divide it into eight subarrays. Each subarray well was treated with 400 µL of ovalbumin (1% wt/vol) dissolved in freshly prepared Hepes blocking buffer ± 500 µM of Zn^2+^ (pH adjusted for individual experiments) for 1 h at ambient temperature in a humid chamber with gentle shaking. Subsequently, the blocking solution was discarded, and a solution of HMGB1 (40 µg/mL) in the same Hepes buffer (± Zn^2+^, defined pH) was added to the subarray. After incubating for 2 h at room temperature with gentle shaking, the slides were extensively washed (first with PBS buffer with 0.1% Tween20 and then with only PBS, pH 7.4) to remove any nonspecific binding. The subarray was further treated with a 1:500 dilution (in PBS) of Cy3-conjugated goat anti-mouse IgG (Fc-specific) secondary antibody and then gently shaken for 1 h in the dark, humid chamber followed by the same washing cycle described earlier. The developed glycan microarray slides were then dried and scanned with a Genepix 4000B (Molecular Devices Corp.) microarray scanner (at 532 nm). Data analysis was performed using the Genepix Pro-7.3 analysis software (Molecular Devices Corp.).

### Purification of HMGB1 from *E. coli* and HEK293 FreeStyle.

Expression and purification of full-length murine His-HMGB1 in *E. coli* were performed as described before (35). Mutagenesis was performed using a QuikChange site-directed mutagenesis kit (Agilent).

For HMGB1 expression in mammalian cells, the complete open reading frame of murine HMGB1 was cloned into pcDNA3.1(+)-C-6His vector (GenScript). Transfection was performed using FectoPRO transfection reagent (Polyplus-transfection). Recombinant His-HMGB1 was produced in 293 FreeStyle cells (Thermo Fisher Scientific). Purification of His-HMGB1 from 293 FreeStyle cell lysate was carried out using Ni Sepharose 6 Fast Flow gel (GE Healthcare). After purification, His-HMGB1 was 99% pure as judged by silver staining.

## Supplementary Material

Supplementary File

## Data Availability

All study data are included in the article and/or *SI Appendix*.
