# Supplementary material for: Sialoglycan recognition is a common connection linking acidosis, zinc, and HMGB1 in sepsis
Source: Proc Natl Acad Sci U S A. 2021 Mar 3;118(10):e2018090118. doi: 10.1073/pnas.2018090118 (PMC7958265; doi:10.1073/pnas.2018090118)

Supplementary Data

for

Sialoglycan Recognition is a Common Connection Linking Acidosis, Zinc and HMGB1 in Sepsis

Shoib S. Siddiqui[1,2*#], Chirag Dhar[1,2*], Venkatasubramaniam Sundaramurthy[1,2##], Aniruddha Sasmal[1,2], Hai Yu[3], Esther Bandala-Sanchez [4,5], Miaomiao Li[6], Xiaoxiao Zhang[6], Xi Chen[3], Leonard C. Harrison[4,5], Ding Xu[6], Ajit Varki[1,2**]

[1]Departments of Medicine and Cellular and Molecular Medicine, [2]Glycobiology Research and Training Center, University of California, San Diego, CA, USA, [3]Department of Chemistry, University of California, Davis, CA, 95616, USA [4]The Walter and Eliza Hall Institute of Medical Research, Parkville, Victoria 3052, Australia, [5]Department of Medical Biology, University of Melbourne, Parkville, Victoria 3010, Australia, [6]Department of Oral Biology, School of Dental Medicine, University at Buffalo, The State University of New York, USA

[#]Current Address: School of Life and Medical Sciences, University of Hertfordshire, College Lane Campus, Hatfield, AL10 9AB, UK.

[# #]Current Address: Department of Molecular and Human Genetics, Baylor College of Medicine, Houston, TX 77030.

*Equal contribution

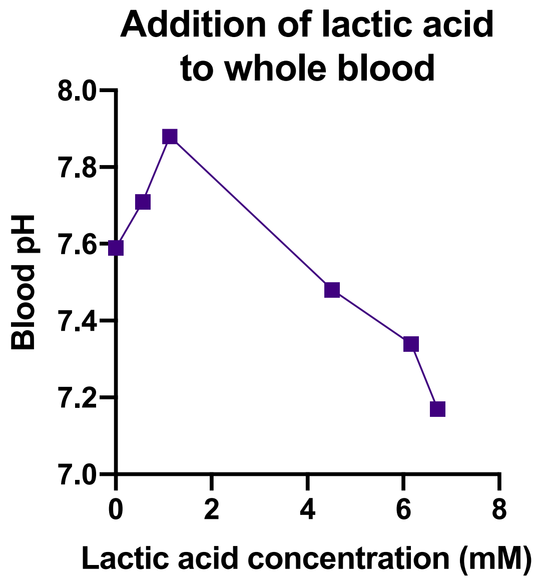

Supplementary Fig 1: Addition of lactic acid to whole blood causes an initial rise and subsequent fall in pH**.**

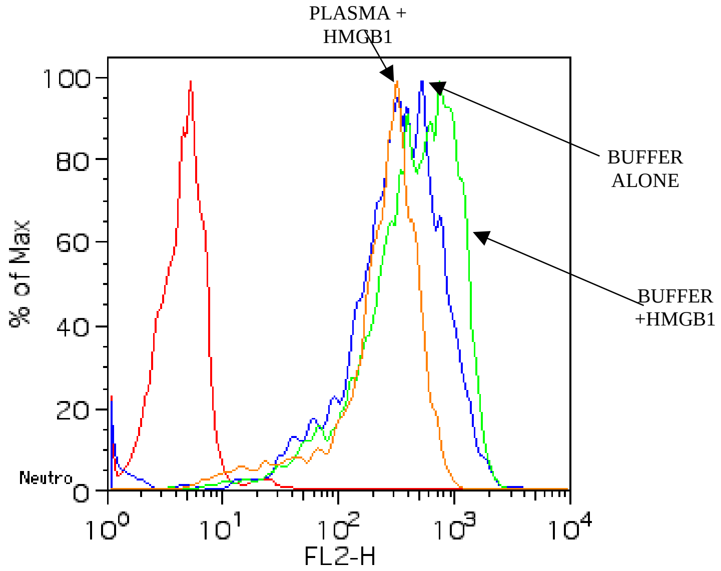

Supplementary Figure 2: Addition of buffer with and without HMGB1. Red chromatogram- secondary control, orange- plasma add back, blue- HEPES buffer with zinc substitution, green- HEPES buffer with zinc and HMGB1 substitution.

Supplementary Fig 3: Previously known binding partner of HMGB1, another anionic glycan heparin does not exhibit this extreme pH sensitivity, and zinc only partially facilitates binding: A) The binding of HMGB1 with heparin was determined by ELISA using a binding buffer at different pH ranges (7.1-7.8) B) The binding assay of HMGB1 and heparin was also performed with a binding buffer with and without zinc. The experiment was performed in triplicate (technical), where data shows mean±SD.

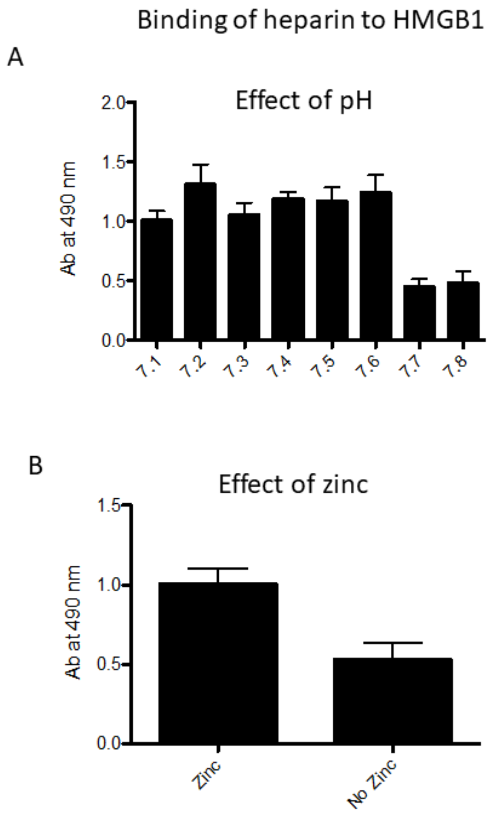

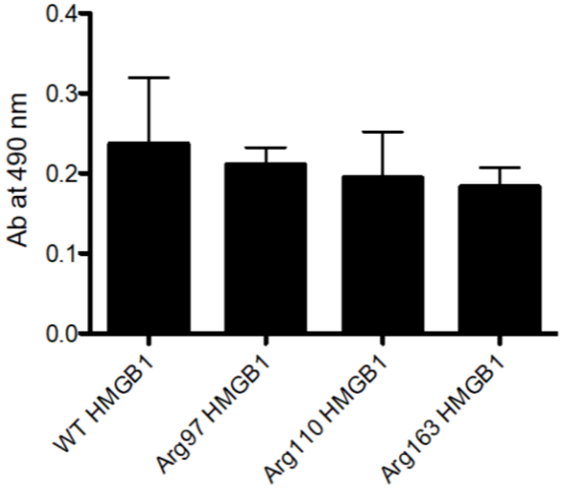

Supplementary Fig 4: Binding of sialic acid with different arginine mutants of HMGB1: Different arginine mutants of HMGB1 B-box were generated and binding of these mutants with 3'- sialyllactose was measured by ELISA (in the presence of 500µM zinc). The experiment was performed in triplicate, where data shows mean±SD. The replicates mentioned here were technical

replicates. There was no statistical significance between different the WT and mutant HMGB1 in their binding abilities to sialic acid.

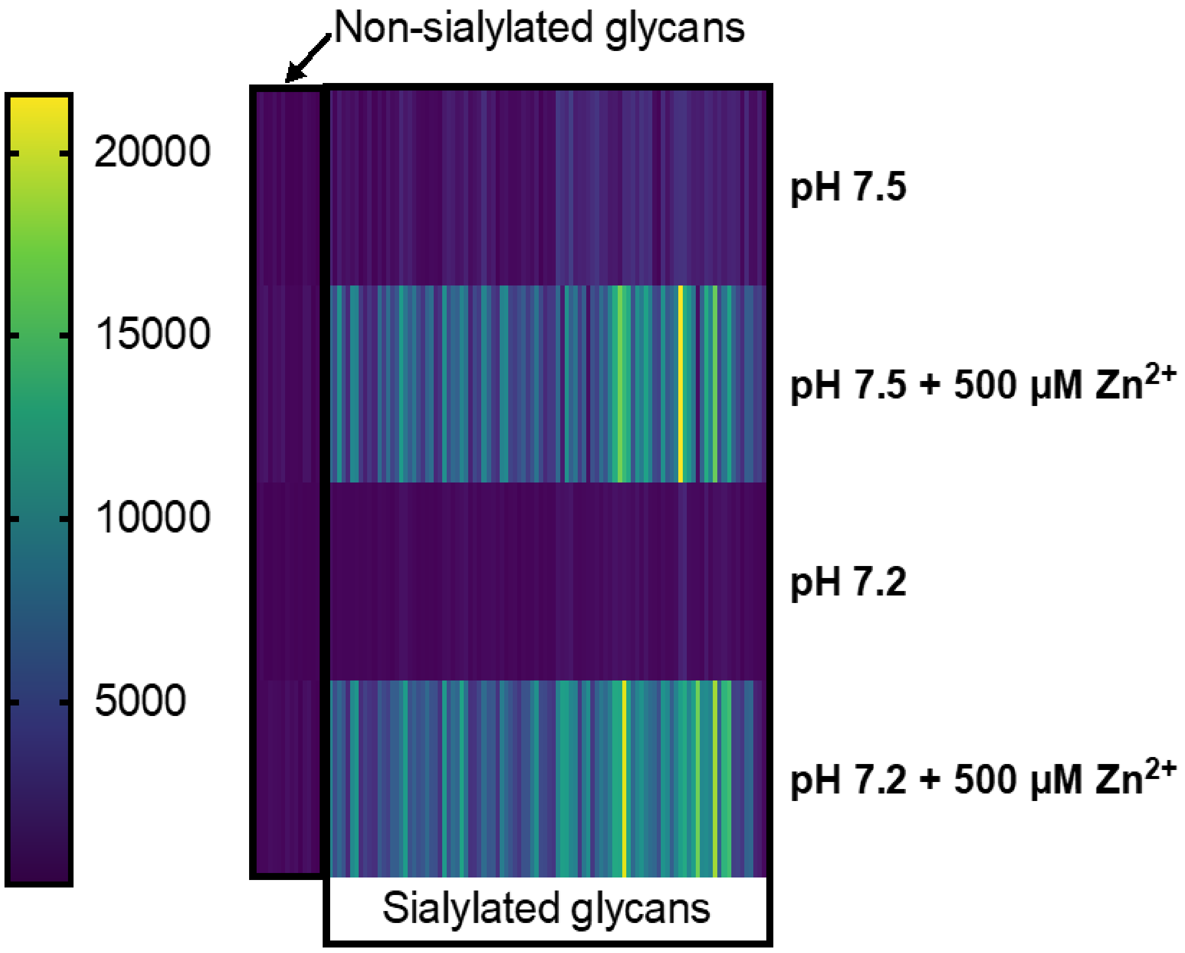

Supplementary Fig 5: Heatmap representing average RFUs of HMGB1 binding to sialosides and non-sialosides under various conditions.

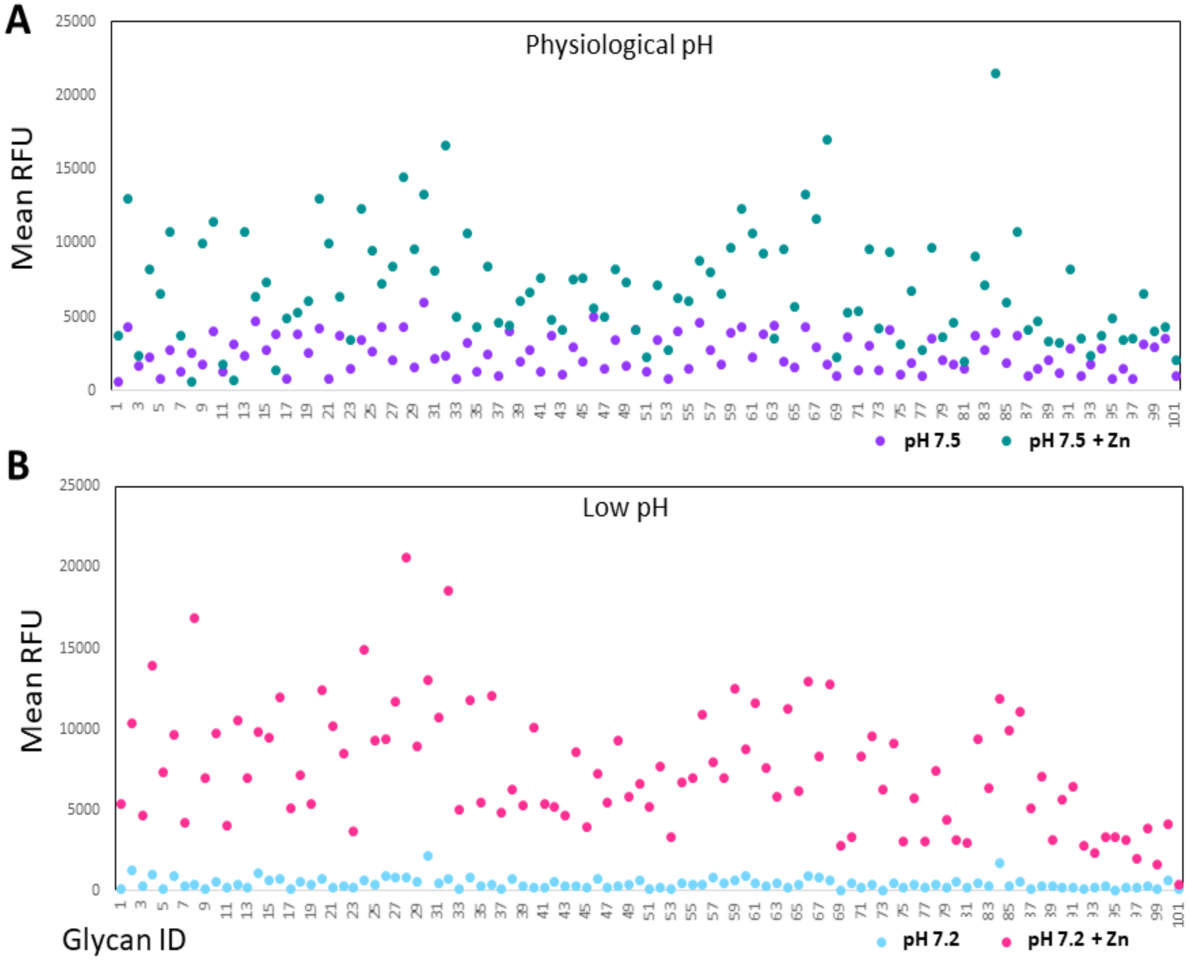

Supplementary Fig 6: Binding of HMGB1 with sialic acids at physiological zinc concentration: The binding of HMGB1 with sialoglycan probes on a glycan array was performed using different zinc concentrations. The data shows mean RFU ± SD.

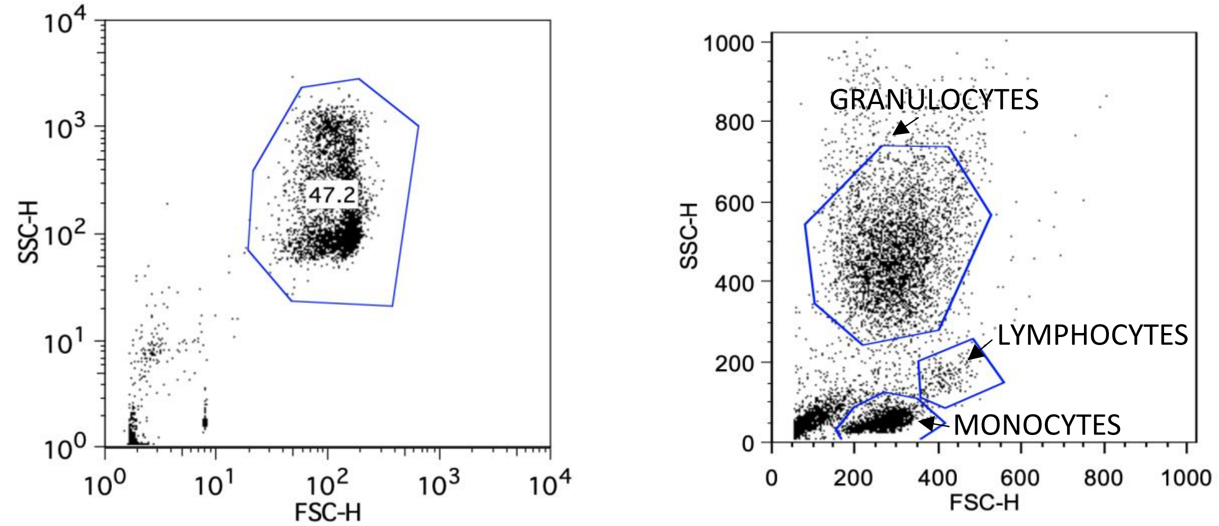

Supplementary figure 7: Whole blood gating strategy. The dot-plot on the left represents erythrocyte population as seen without RBC lysis while the right represents granulocytes, monocytes and lymphocytes as seen in RBC lysed blood.

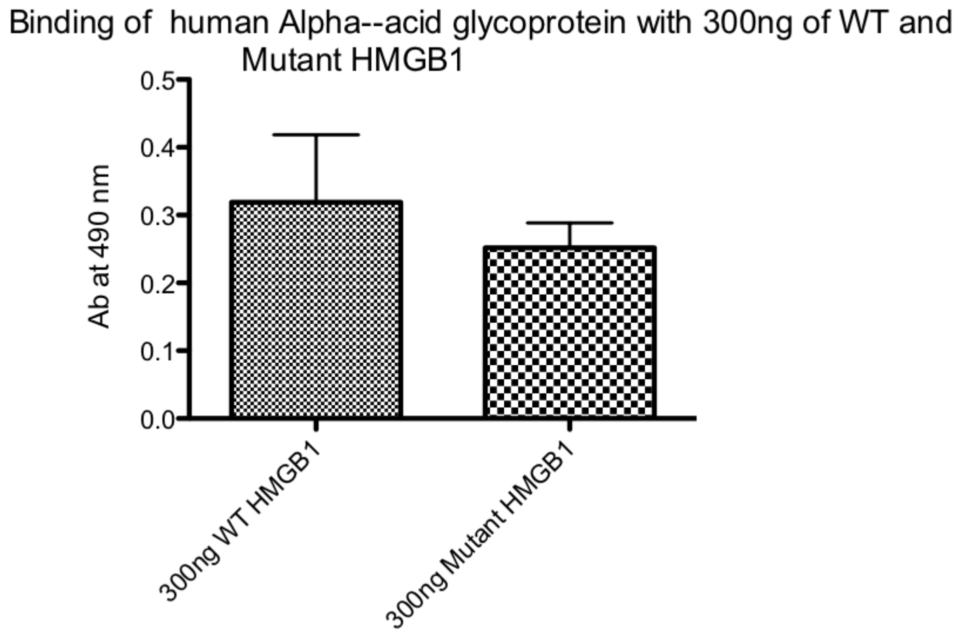

Supplementary Figure 8: The binding of WT and mutant HMGB1 with α-1-acid glycoprotein: In this ELISA the WT HMGB1 and HS-binding deficient quintuple mutant of HMGB1 was used to check their binding to α-1-acid glycoprotein. The experiments were performed in triplicates which were technical replicates, n=3. The data shows Mean±SD.

Supplementary Table 1: Glycan list- HMGB1

Glycan ID	Glycan Structure
46	Galβ-NH2
48	GalβR2
49	GalβR1
78	Galα3Galβ4GlcNAcβR1
52	Galβ3GalNAcαR1
51	Galβ3GalNAcβR1
53	Galβ3GlcNAcβR1
43	Galβ4GlcβR1
44	Galβ4Glcβ-NH2
50	Galβ4GlcβR2
45	Galβ4GlcNAcβR1
89	Galβ4GlcNAcβ3Galβ4GlcβR1
54	Galβ4GlcNAc6SβR1
242	(GalNAcβ4)Galβ4GlcβR1
83	Gal6Sβ4(Fucα3)GlcNAcβR1
86	Gal6Sβ4(Fucα3)GlcNAc6SβR1
47	GalNAcαR1
25	Neu5Acα3GalβR1
15	Neu5Acα3Galβ3GalNAcαR1
33	Neu5Acα3Galβ3GalNAcβR1
13	Neu5Acα3Galβ3GlcNAcβR1
60	Neu5Acα3Galβ3GlcNAcβ3Galβ4GlcβR1
238	Neu5Acα3Galβ3(Fucα4)GlcNAcβR1
21	Neu5Acα3Galβ4GlcβR1
80	Neu5Acα3Galβ4GlcβR2
126	Neu5Acα3Galβ4GlcβR5
11	Neu5Acα3Galβ4GlcNAcβR1
123	Neu5Acα3Galβ4GlcNAcβR5
90	Neu5Acα3Galβ4GlcNAcβ3Galβ4GlcβR1
62	Neu5Acα3Galβ4GlcNAc6SβR1
55	Neu5Acα3Galβ4(Fucα3)GlcNAcβR1
57	Neu5Acα3Galβ4(Fucα3)GlcNAc6SβR1
96	Neu5Acα3(β4GalNAc)Galβ4GlcβR1
84	Neu5Acα3Gal6Sβ4(Fucα3)GlcNAcβR1

87	Neu5Acα3Gal6Sβ4(Fucα3)GlcNAc6SβR1
27	Neu5Acα6GalβR1
19	Neu5Acα6Galβ4GlcβR1
125	Neu5Acα6Galβ4GlcβR5
17	Neu5Acα6Galβ4GlcNAcβR1
124	Neu5Acα6Galβ4GlcNAcβR5
5	Neu5Acα6GalNAcαR1
41	Neu5Acα8Neu5Acα3Galβ4GlcβR1
64	Neu5Acα8Neu5Acα3Galβ4GlcβR4
98	Neu5Acα8Neu5Acα3(β4GalNAc)Galβ4GlcβR1
76	Neu5Acα8Neu5Acα6Galβ4GlcβR1
42	Neu5Acα8Neu5Acα8Neu5Acα3Galβ4GlcβR1
65	Neu5Acα8Neu5Acα8Neu5Acα3Galβ4GlcβR4
72	Neu5Acα8Neu5Gcα3Galβ4GlcβR1
73	Neu5Acα8Neu5Gcα6Galβ4GlcβR1
71	Neu5Acα8Kdnα6Galβ4GlcβR1
104	Neu4,5Ac2α3Galβ3GalNAcαR1
103	Neu4,5Ac2α3Galβ3GalNAcβR1
102	Neu4,5Ac2α3Galβ3GlcNAcαR1
101	Neu4,5Ac2α3Galβ3GlcNAcβR1
99	Neu4,5Ac2α3Galβ4GlcβR1
79	Neu4,5Ac2α3Galβ4GlcNAcβR1
100	Neu4,5Ac2α3Galβ4GlcNAcβ3Galβ4GlcβR1
29	Neu5,9Ac2α3GalβR1
9	Neu5,9Ac2α3Galβ3GalNAcαR1
35	Neu5,9Ac2α3Galβ3GalNAcβR1
7	Neu5,9Ac2α3Galβ3GlcNAcβR1
240	Neu5,9Ac2α3Galβ3(Fucα4)GlcNAcβR1
39	Neu5,9Ac2α3Galβ4GlcβR1
1	Neu5,9Ac2α3Galβ4GlcNAcβR1
92	Neu5,9Ac2α3Galβ4GlcNAcβ3Galβ4GlcβR1
31	Neu5,9Ac2α6GalβR1
37	Neu5,9Ac2α6Galβ4GlcβR1
3	Neu5,9Ac2α6Galβ4GlcNAcβR1
23	Neu5,9Ac2α6GalNAcαR1
243	Neu5,9Ac2a8Neu5Acα3Galβ4GlcβR1
26	Neu5Gcα3GalβR1

16	Neu5Gcα3Galβ3GalNAcαR1
34	Neu5Gcα3Galβ3GalNAcβR1
14	Neu5Gcα3Galβ3GlcNAcβR1
61	Neu5Gcα3Galβ3GlcNAcβ3Galβ4GlcβR1
239	Neu5Gcα3Galβ3(Fucα4)GlcNAcβR1
22	Neu5Gcα3Galβ4GlcβR1
12	Neu5Gcα3Galβ4GlcNAcβR1
91	Neu5Gcα3Galβ4GlcNAcβ3Galβ4GlcβR1
63	Neu5Gcα3Galβ4GlcNAc6SβR1
56	Neu5Gcα3Galβ4(Fucα3)GlcNAcβR1
58	Neu5Gcα3Galβ4(Fucα3)GlcNAc6SβR1
97	Neu5Gcα3(β4GalNAc)Galβ4GlcβR1

85	Neu5Gcα3Gal6Sβ4(Fucα3)GlcNAcβR1
88	Neu5Gcα3Gal6Sβ4(Fucα3)GlcNAc6SβR1
28	Neu5Gcα6GalβR1
20	Neu5Gcα6Galβ4GlcβR1
18	Neu5Gcα6Galβ4GlcNAcβR1
6	Neu5Gcα6GalNAcαR1
69	Neu5Gcα8Neu5Acα3Galβ4GlcβR1
75	Neu5Gcα8Neu5Gcα3Galβ4GlcβR1
77	Neu5GcMeα8Neu5Acα3Galβ4GlcβR1
111	Neu4Ac5Gcα3Galβ3GalNAcαR1
110	Neu4Ac5Gcα3Galβ3GalNAcβR1
109	Neu4Ac5Gcα3Galβ3GlcNAcαR1
108	Neu4Ac5Gcα3Galβ3GlcNAcβR1
106	Neu4Ac5Gcα3Galβ4GlcβR1
105	Neu4Ac5Gcα3Galβ4GlcNAcβR1
107	Neu4Ac5Gcα3Galβ4GlcNAcβ3Galβ4GlcβR1
30	Neu5Gc9Acα3GalβR1
10	Neu5Gc9Acα3Galβ3GalNAcαR1
36	Neu5Gc9Acα3Galβ3GalNAcβR1
8	Neu5Gc9Acα3Galβ3GlcNAcβR1
40	Neu5Gc9Acα3Galβ4GlcβR1
2	Neu5Gc9Acα3Galβ4GlcNAcβR1
93	Neu5Gc9Acα3Galβ4GlcNAcβ3Galβ4GlcβR1
32	Neu5Gc9Acα6GalβR1
38	Neu5Gc9Acα6Galβ4GlcβR1
4	Neu5Gc9Acα6Galβ4GlcNAcβR1

24	Neu5Gc9Acα6GalNAcαR1
245	Neu5Acα3(Galβ3GalNAcβ4)Galβ4GlcβR1
247	Neu5Acα3(Fuca2Galβ3GalNAcβ4)Galβ4GlcβR1
66	Neu5Acα6(Neu5Acα3)Galβ4GlcβR1
67	Neu5Acα6(Neu5Gcα3)Galβ4GlcβR1
68	Neu5Acα6(Kdnα3)Galβ4GlcβR1
244	Neu5Aca8Neu5Acα3(Galβ3GalNAcβ4)Galβ4GlcβR1
246	Neu5Gcα3(Galβ3GalNAcβ4)Galβ4GlcβR1
248	Neu5Gcα3(Fuca2Galβ3GalNAcβ4)Galβ4GlcβR1

R1 = Pro-NH_2

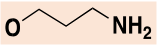

R2 = HEG-NH_2

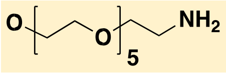

R4 = Pro-HEG-NH_2

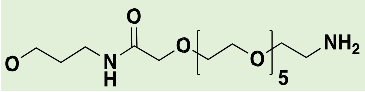

R5 = Pro-Htrz-TEG-NH_2

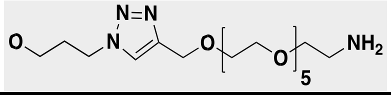

Supplement: Supplementary File [file pnas.2018090118.sapp.pdf]
